# Therapeutic effects of coronary granulocyte colony-stimulating factor on rats with chronic ischemic heart disease

**DOI:** 10.1515/biol-2020-0078

**Published:** 2020-10-20

**Authors:** Pengcheng Ren, Ming Zhang, Shuren Dai

**Affiliations:** Department of Cardiology, Chongqing Dazu District People’s Hospital, Chongqing, 402360, People's Republic of China

**Keywords:** myocardial ischemia, granulocyte colony-stimulating factor, ventricular function, apoptosis

## Abstract

**Background:**

The aim of this study was to evaluate the therapeutic effects of coronary granulocyte colony-stimulating factor (G-CSF) on rats with chronic ischemic heart disease (CIHD).

**Methods:**

Thirty healthy rats were randomly divided into control, subcutaneous and intracoronary G-CSF injection groups (*n* = 10) after the CIHD model was established. Left ventricular ejection fraction (LVEF), myocardial injury area, myocardial perfusion area and viable myocardium were observed by coronary angiography, dual-isotopic myocardial imaging and first-pass delayed myocardial perfusion magnetic resonance imaging (MRI) before modeling as well as 2 and 4 weeks after surgery.

**Results:**

The peak times of peripheral blood and subcutaneous G-CSF levels were 3 and 5 days after mobilization, respectively. The peripheral blood CD34+/CD133+ cell ratio of subcutaneous or intracoronary G-CSF injection group significantly exceeded that of the control group (*P* < 0.05). The distal stenosis degrees of target lesions in subcutaneous and intracoronary G-CSF injection groups were significantly lower than that of the control group (*P* < 0.05). Compared with the situation before mobilization, LVEF was significantly improved after 2 weeks in intracoronary and subcutaneous G-CSF injection groups (*P* < 0.01). Their infarcted myocardial areas were reduced, the left ventricular remodeling was relieved, the percentage of viable myocardium was increased, angiogenesis was promoted and cardiomyocyte apoptosis was inhibited.

**Conclusion:**

Intracoronary G-CSF injection is safe and effective as subcutaneous injection, improving the cardiac function of CIHD rats.

## 1 Introduction

Regardless of advances in drug therapy and surgical procedures, patients with chronic ischemic heart disease (CIHD) often die of heart failure [1]. In recent years, it has been well documented that stem cell transplantation can improve cardiac function and prevent ventricular remodeling in CIHD patients, thus improving their prognosis [2]. Granulocyte colony-stimulating factor (G-CSF) can mobilize bone marrow stem cells into the peripheral blood, and spontaneous homing upon myocardial infarction or myocardial ischemia contributes to the migration of mobilized stem cells to infarcted myocardium-related areas [3]. As G-CSF treatment is noninvasive, it may be more applicable for the treatment of myocardial infarction and ischemic heart disease [4]. Previous animal studies focused mostly on acute myocardial infarction. However, there are few relevant studies for patients with chronic myocardial ischemia or old myocardial infarction, and the results are inconsistent [5]. Moreover, subcutaneous injection in clinical practice usually requires continuous injection for 3–7 consecutive days and even more than 10 days, and a large amount of G-CSF injection may result in hypercoagulable blood, increasing the risk of recurrent angina or myocardial infarction [6]. Therefore, we intended to study whether other injection routes can be used to reduce the amount of G-CSF while improving the cardiac function, which, in turn, may reduce adverse reactions after G-CSF mobilization.

The aim of this study was to compare the effects of intracoronary injection and subcutaneous injection of G-CSF on the cardiac function and the left ventricular remodeling in rats with CIHD and to evaluate its safety. The results provide valuable experimental evidence for clinical application.

## 2 Materials and methods

### 2.1 Materials and apparatus

Thirty healthy Sprague Dawley rats weighing 200–220 g of either gender were purchased from SLAC Laboratory Animals Co., Ltd [Shanghai, China; license number: SCXK (Shanghai) 2018-0008].

Recombinant human G-CSF was purchased from Harbin Pharmaceutical Group Co., Ltd (China; batch number: 20180816G). G-CSF ELISA kit and TUNEL DeadEnd kit were bought from Santa Cruz (USA). Fluorescein isothiocyanate (FITC)-labeled monoclonal mouse antihuman CD34 antibody and APC-labeled monoclonal mouse antihuman CD133 antibody were obtained from Amresco (USA). TNF-α, cTnT and BNP ELISA kits, reverse transcription system and Access RT-PCR kits as well as ECL Plus kit were provided by Sigma (USA). Goat antimouse Akt1 and vWF polyclonal antibodies were purchased from Shanghai Baoman Biotechnology Co., Ltd (China). Magnevist (Gd-DTPA) injection was bought from Schering AG (Germany; batch number: M33261). Omnipaque (iohexol) injection was obtained from GE Healthcare (Shanghai, China; batch number: 20180325). 99mTc-MIBI injection was provided by Guangzhou Medical Isotope Service Center (China; batch number: 20180122). All other reagents were analytically pure.

Ameroid constrictor rings were purchased from Labnet (USA). PCR system and FACSCalibur flow cytometer were bought from Thermo Fisher Scientific (USA). Inverted fluorescent microscope and image analysis system were obtained from Tohnichi (Japan). Fluorescent imaging system was provided by Medica (USA). Innova 2000 Digital Cardiac Cath Lab System was purchased from GE Healthcare (Shanghai, China).

### 2.2 Model establishment

Left thoracotomy was performed under sterile conditions, and the free circumflex artery was about 1.0–1.5 cm. An ameroid constrictor ring of suitable size was quickly placed on the artery outside the vascular cavity. After surgery, aspirin (100 mg/day) was administered orally until death. The model establishment was considered successful, if two or more of the following four conditions were met: (1) ST segment depression and/or *T* wave changes (from elevation to inversion or inversion to elevation) in leads *I*, aVL and (or) II, III, aVF and/or V4 to V6 found on ECG; (2) coronary angiography showing subtotal or complete occlusion of left circumflex (LCX) branch (stenosis > 95%); (3) cardiac MRI showing perfusion defects and/or dyskinesia of left ventricular lateral wall; (4) myocardial perfusion imaging of single-photon emission computed tomography (SPECT) suggesting perfusion defects and/or metabolic/perfusion mismatch of the left ventricular lateral wall [7].


**Ethical approval:** The research related to animal use has been complied with all the relevant national regulations and institutional policies for the care and use of animals.

### 2.3 Grouping and administration

One week after successful modeling, 30 otherwise healthy rats were randomly divided into control group, subcutaneous G-CSF injection group and intracoronary G-CSF injection group (*n* = 10). G-CSF was administered once into the coronary artery at 50 μg/kg for the intracoronary injection group, and 10 µg/kg/day for the subcutaneous injection group for 7 consecutive days. The control group was untreated.

### 2.4 Imaging examination

The vital signs of all rats were recorded before and 2 weeks after model establishment, and coronary angiography, 99mTc-methoxyisobutyl isocyanide/fluoro-18 fluorodeoxyglucose dual-isotopic myocardial imaging [99mTc-MIBI/18 F-FDG dual-isotope simultaneous acquisition (DISA) SPECT] and cardiac magnetic resonance imaging (MRI) were performed. Coronary angiography data were analyzed by a specialist who did not know the grouping, and computerized quantitative analysis (QCA) was made according to the standard method on the minimum lumen diameter of the vessel proximal to the constrictor ring, reference lumen diameter and diameter stenosis, while the collateral branches of coronary arteries were classified according to the Rentrop collateral circulation classification method. The data of myocardial radionuclide scans were analyzed by ECTb 3.0 software to determine the defect area of the left ventricular myocardial perfusion and viable myocardium. The percentage of myocardial perfusion defect area of the left ventricle (%) = (*M*_d_/*M*_t_) × 100%, where *M*_d_ is the myocardial mass at perfusion defect and *M*_t_ is the entire myocardial mass. The percentage of viable myocardium = (metabolic/perfusion mismatch area)/(metabolic/perfusion mismatch area + metabolic/perfusion match area) × 100%, where metabolic/perfusion mismatch area represents viable myocardium, and metabolic/perfusion match area represents nonviable myocardium. MRI images were analyzed by computer software using magnetic resonance specialists to determine left ventricular ejection fraction (LVEF), left ventricular end-diastolic volume (LVEDV), left ventricular end-systolic volume (LVESV) and left ventricular myocardial infarct area.

### 2.5 Detection of biomarkers

In the intracoronary G-CSF injection group, 10 mL of venous blood was collected from rats before model establishment, 1, 2, 3, 5 and 7 days after G-CSF coronary injection and before execution.

#### 2.5.1 Flow cytometry

CD34+ cell count and CD34+/CD133+ cell ratio were detected by flow cytometry as follows [8]. Anticoagulated whole blood (50 µL) was divided into blank control, anti-CD34-PE, anti-CD34-FITC and anti-CD133-APC groups. After the addition of antibody into each tube, the mixture was incubated in a 4°C refrigerator in the dark for 30 min, added 1 mL of diluted erythrocyte lysis buffer, fully pipetted, placed in a 37°C incubator in the dark for 10 min and centrifuged at 1,500 rpm for 5 min at room temperature. After the supernatant was discarded, the residue was washed with 2 mL of phosphate-buffered saline (PBS) and centrifuged at 1,500 rpm for 5 min at room temperature. Then, the supernatant was discarded, and the residue was mixed with 0.5 mL of PBS and subjected to flow cytometry to count positive cells.

#### 2.5.2 Enzyme-linked immunosorbent assay (ELISA)

The serum levels of G-CSF (G-CSF ELISA kit; Santa Cruz Biotechnology, USA), lactic dehydrogenase (LDH) (LDH ELISA kit; Sigma, USA), TNF-α (TNF-α ELISA kit; Sigma, USA), cardiac troponin T (cTnT) (cTnT ELISA kit; Sigma, USA) and type B natriuretic peptide (BNP) (BNP ELISA kit; Sigma, USA) were determined by ELISA.

Briefly, capture antibody was 1:200 diluted, added to a 96-well plate at 100 µL/well and coated overnight at 4°C. After capture antibody was shaken off, the plate was washed with 1× washing buffer (100 µL/well) four times, 1 min each time and dried on absorbent paper the last time. After blocking with 100 µL/well of blocking solution for 2 h at room temperature, the plate was washed four times with 1× washing buffer (100 µL/well), 1 min each time and dried on absorbent paper the last time. The standard was diluted into six concentrations, and each concentration had one replicate. Then, the blocking solution was shaken off, and the plate was washed twice. Blank well was added 100 µL of diluent, standard wells were added standards of each concentration, and 100 μL of supernatant was added to sample wells. Afterward, 100 µL/well of detection antibody was added to each well and incubated at room temperature for 2 h. Then, the liquid was shaken off, and the plate was washed four times. Subsequently, 100 µL/well of reaction solution was added, incubated at room temperature for 10 min and sealed with parafilm. Then, the liquid was shaken off, and the plate was washed four times. The optical density of each group was detected by a microplate reader 40 min after the color development reagent at 100 µL/well was added. Standard curves were thereafter plotted to calculate the corresponding concentrations.

#### 2.5.3 Hematoxylin and eosin (HE) staining

After 2 weeks of G-CSF treatment, all rats were executed to separate the left anterior descending branch, LCX branch and their corresponding area of donor myocardium. HE staining was performed for the myocardium, liver, lung, kidney, spleen and lymph nodes.

Paraffin sections were baked in a 60°C oven for 1 h, deparaffinized with xylene I and II solutions for 10 min each, hydrated with series concentrations of ethanol solutions until water and washed with PBS for 3 min. Afterward, they were stained with hematoxylin for 10 min, rinsed with tap water for 10 min, differentiated with 1% HCl-ethanol solution for 10 s, rinsed with tap water for 10 min, stained with eosin for 3 min and rinsed with tap water and distilled water for 30 s each. Subsequently, the sections were dehydrated with series concentrations of ethanol solutions, transparentized with xylene I and II solutions for 10 min each, mounted with neutral resin, observed under a microscope and analyzed with Image-Pro Plus 6.0 software.

#### 2.5.4 TUNEL assay

TUNEL assay was used to detect the apoptosis of myocardial cells in the infarcted marginal area, local neovascularization and expression levels of factors related to apoptosis and angiogenesis.

The sections were baked in a 60°C oven for 10 min, deparaffinized by using xylene twice, hydrated with series concentrations of ethanol solutions until water and washed with PBS three times, 1 min each time. After antigen retrieval by using 200 mL of 0.1 M citrate buffer (pH 6.0), the sections were blocked at room temperature for 10 min and washed with PBS three times, 1 min each time. Then, the TUNEL solution comprising 45 μL of label solution and 5 μL of enzyme solution was dropped onto the sections (50 μL each) for incubation at 37°C for 60 min. Subsequently, the sections were washed with PBS twice, 2 min each time, and the cell nuclei were stained with 0.5 μg/mL 4′,6-diamidino-2-phenylindole (DAPI) solution at room temperature. The liquid was shaken off, and the sections were washed with PBS, dropped antiquenching reagent, covered with coverslips and observed under a fluorescence microscope.

#### 2.5.5 Western blotting

The expression level of vWF protein at the marginal area of infarcted myocardium was detected by Western blotting as follows [9]. A small amount of myocardial tissue was minced, homogenized by using lysis buffer containing phenylmethylsulfonyl fluoride (PMSF) and centrifuged to obtain the supernatant. The protein concentration was measured with bicinchoninic acid (BCA) kit, diluted, added 5× loading buffer and denatured for 5 min. The amount of sample loading was 160 µg per well (*n* = 3). Proteins were separated with 10% SDS-PAGE, and the products were transferred to a nitrocellulose membrane. Subsequently, the membrane was blocked with 5% skim milk overnight at 4°C, incubated with primary antibody against vWF (1:500) overnight at 4°C and then with horseradish peroxidase (HRP)-conjugated secondary antibody (1:5,000) at room temperature for 2 h and exposed to a gel imaging system. Finally, the grayscale values of protein bands were analyzed.

### 2.6 Statistical analysis

All data were statistically analyzed by SPSS 13.0 software. Continuous variables were expressed as (mean ± standard deviation). The comparisons of numerical variables between groups were conducted by analysis of variance. For pairwise comparisons, the Bonferroni method was used in the case of homogeneity of variance. If there was no homogeneity of variance, Dunnett’s method was employed. The paired *t* test was carried out for the same continuous variable at the beginning and after follow-up. Categorical variables were compared with the *χ* ^2^ test, and Fisher’s exact test was performed when the sample size was small. *P* < 0.05 was considered statistically significant.

## 3 Results

### 3.1 General state

No death occurred during the establishment of the CIHD model. After randomization, no adverse events such as death, myocardial infarction, infection and heart failure occurred.

### 3.2 Serum G-CSF level detected by ELISA

**Figure 1:**
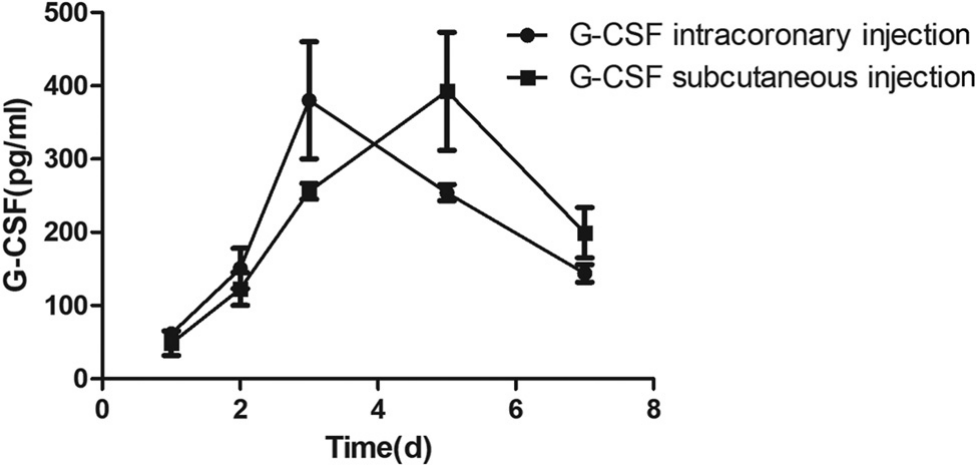
Serum G-CSF levels of two groups at different time points.

After intracoronary injection of G-CSF, the peak time of G-CSF level in the peripheral blood of rats with CIHD was 3 days after G-CSF mobilization, while the peak of subcutaneous G-CSF level appeared 5 days after mobilization, but no significant difference was found between those of the former two groups [(381.2 ± 79.6) pg/mL vs (392.4 ± 80.4) pg/mL, *P* > 0.05] (Figure 1).

### 3.3 CD34+ cell count and CD34+/CD133+ cell ratio

**Figure 2:**
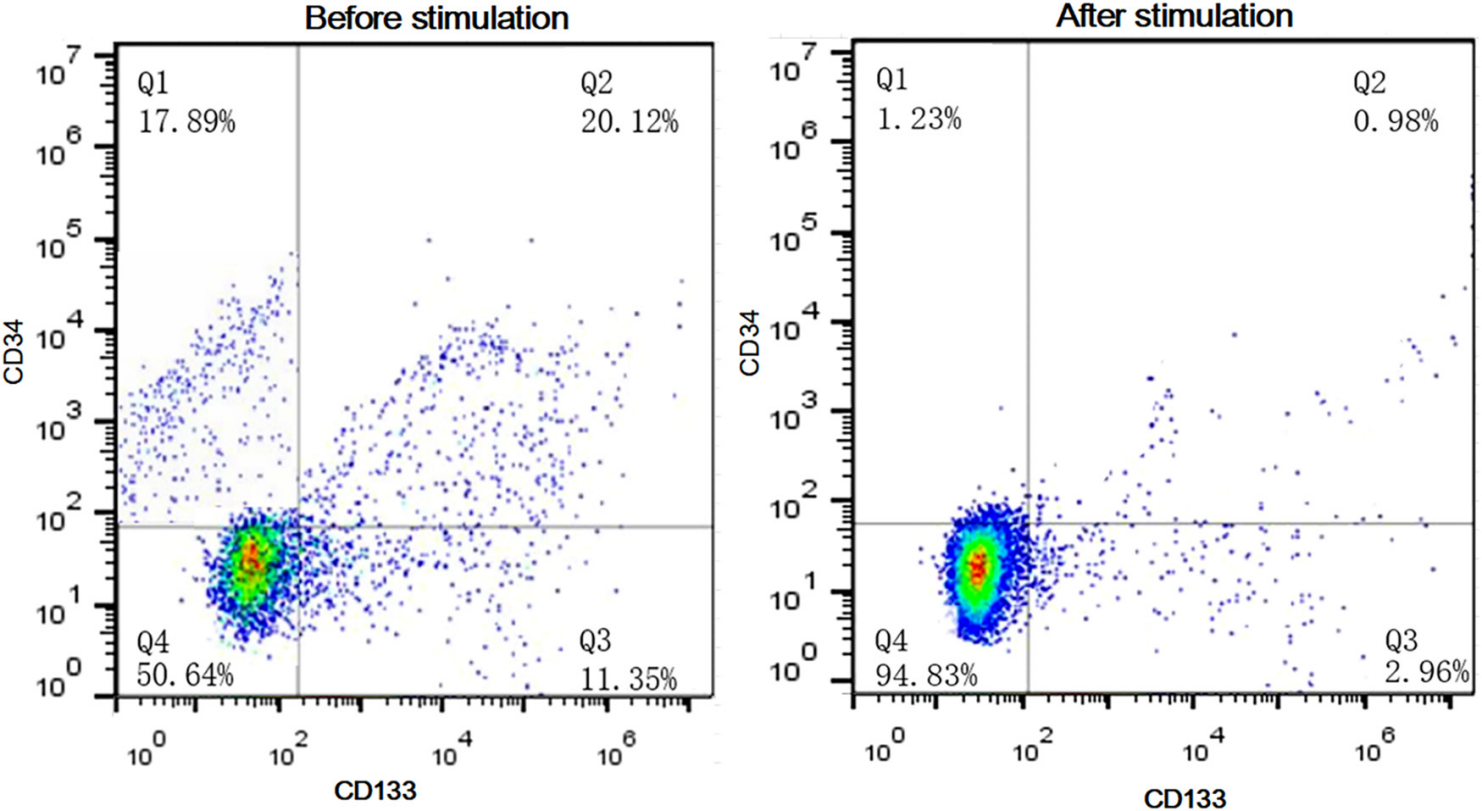
CD34+ cell count and CD34+/CD133+ cell ratio changes in the peripheral blood before and after modeling. Q1: CD34+ cells; Q2: CD34+/CD133+ cells; Q3: CD133+ cells; Q4: CD34−/CD133− cells.

CD34+ cell count and CD34+/CD133+ cell ratio significantly decreased after modeling compared with those before modeling. After 2 weeks of G-CSF injection, the ratios of CD34+ cells in the peripheral blood of the control group, the intracoronary G-CSF injection group and the subcutaneous G-CSF injection group were (1.2 ± 0.4)%, (18.2 ± 3.1)% and (21.6 ± 3.3)%, respectively. The CD34+/CD133+ cell ratios were (1.3 ± 0.3)%, (17.8 ± 2.9)% and (20.9 ± 2.8)%, respectively, suggesting that different routes of G-CSF injection increased such ratios after 2 weeks. However, there was no significant difference between the two treatment groups (*P* > 0.05) (Figure 2).

### 3.4 Degree of LCX artery stenosis and determination of collateral circulation

Four weeks after surgery, the degree of distal LCX stenosis was slightly aggravated in the control group [(27.7 ± 2.5)% vs (25.4 ± 2.3)%, *P* > 0.05]. In the subcutaneous G-CSF injection group and intracoronary G-CSF injection group, the stenosis degree of LCX artery significantly decreased [(16.8 ± 1.6)% vs (26.9 ± 2.1)% and (17.0 ± 1.8)% vs (27.3 ± 2.0)%, *P* < 0.05]. However, the difference between the two treatment groups was not statistically significant (*P* > 0.05). Compared with the postoperative 2nd week, the degree of proximal vascular stenosis decreased in the subcutaneous G-CSF injection group and intracoronary G-CSF injection group in the postoperative 4th week (*P* < 0.05). Also, there was no significant difference between the two treatment groups. Eight weeks after surgery, no collateral vessel was formed in the control group, and one animal in the subcutaneous G-CSF injection group and intracoronary G-CSF injection group each had the vessels (Table 1).

**Table 1:** QCA measurement results of proximal vessels of constrictor ring

	Control group	Subcutaneous G-CSF injection group	Intracoronary G-CSF injection group
**Before surgery**			
Reference lumen diameter (mm)	1.08 ± 0.04	1.09 ± 0.03	1.05 ± 0.04
Ameroid constrictor ring diameter (mm)	1.10 ± 0.06	1.07 ± 0.04	1.11 ± 0.05
**2 weeks after surgery**			
Reference lumen diameter (mm)	1.09 ± 0.05	1.10 ± 0.04	1.08 ± 0.05
Minimum lumen diameter (mm)	0.93 ± 0.04	0.95 ± 0.05	0.94 ± 0.05
Luminal stenosis percentage (%)	14.67 ± 2.13	13.64 ± 2.42	12.96 ± 2.17
**4 weeks after surgery**			
Reference lumen diameter (mm)	1.08 ± 0.06	1.09 ± 0.05	1.07 ± 0.04
Minimum lumen diameter (mm)	0.79 ± 0.04*	0.88 ± 0.04*^#^	0.86 ± 0.05*^#^
Luminal stenosis percentage (%)	26.85 ± 3.11*	19.26 ± 2.71*^#^	19.63 ± 2.65*^#^

*P < 0.05 compared with postoperative 2nd week; ^#^ *P* < 0.05 compared with the control group.

### 3.5 Effects of different G-CSF treatments on left ventricular function and remodeling of CIHD rats assessed by SPECT and MRI

**Figure 3:**
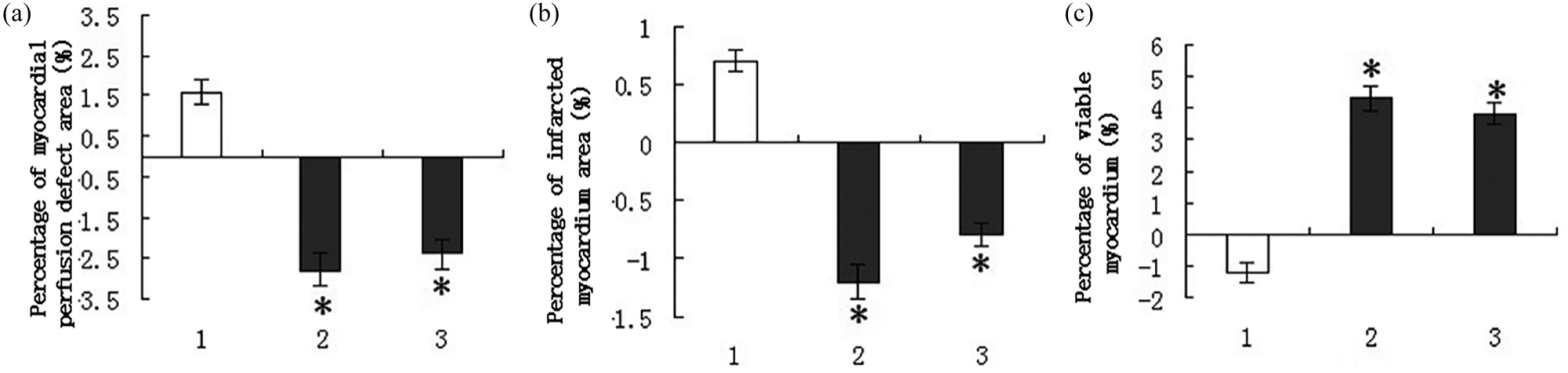
Myocardial perfusion defect area, myocardial infarct size and the percentage of viable myocardium of CIHD rats after different treatments. (a) SPECT evaluation of myocardial perfusion defect area. (b) MRI evaluation of myocardial infarct size. (c) DISA-SPECT evaluation for the percentage of viable myocardium. (1) Control group, (2) subcutaneous G-CSF injection group and (3) intracoronary G-CSF injection group. *P < 0.05 compared with the control group.

SPECT and MRI showed that compared with the postoperative 2nd week, LVEFs of both intracoronary G-CSF injection group and subcutaneous G-CSF injection group were significantly improved in the postoperative 4th week (*P* < 0.01) (Table 2). The improvement of LVEF of the intracoronary G-CSF injection group and subcutaneous G-CSF injection group was more obvious than that of the control group [(−1.1 ± 0.2)%, (2.3 ± 0.4)% vs (2.1 ± 0.3)%, *P* < 0.01]. Nevertheless, the difference between the two routes was not statistically significant (*P* > 0.05). Besides, although the difference in LVEDV between the intracoronary G-CSF injection group and the subcutaneous G-CSF injection group was not statistically significant 4 weeks after surgery, the changes exceeded that of the control group (*P* < 0.05). The LVESV reduction of the intracoronary G-CSF injection group and the subcutaneous G-CSF injection group exceeded that of the control group, but there was no significant difference between the former two groups, suggesting that both administration routes can improve LVEF of CIHD rats and inhibit left ventricular remodeling. Additionally, the myocardial perfusion defect area and myocardial infarct size of the intracoronary G-CSF injection group and subcutaneous G-CSF injection group were also smaller than those of the control group 8 weeks after surgery (*P* < 0.05), but the difference between the former two groups was not statistically significant (Figure 3a and b). The percentage of viable myocardium in the control group was significantly lower than in the intracoronary G-CSF injection group and subcutaneous G-CSF injection group (*P* < 0.05) (Figure 3c).

**Table 2:** MRI evaluation of left ventricular function

	Control group	Subcutaneous G-CSF injection group	Intracoronary G-CSF injection group
**2 weeks after surgery**			
LVEF (%)	26.3 ± 1.4	26.1 ± 1.2	27.5 ± 1.3
LVEDV (mL)	18.5 ± 1.3	18.7 ± 1.3	18.4 ± 1.2
LVESV (mL)	6.9 ± 0.8	7.1 ± 0.7	6.8 ± 0.8
**4 weeks after surgery**			
LVEF (%)	25.5 ± 1.4*	29.0 ± 1.5*^#^	29.5 ± 1.4*^#^
LVEDV (mL)	18.8 ± 1.3	18.4 ± 1.4*	18.2 ± 1.3*
LVESV (mL)	7.5 ± 0.8*	6.4 ± 0.7*^#^	6.3 ± 0.7*^#^

LVEF: left ventricular ejection fraction; LVEDV: left ventricular end diastolic volume; LVESV: left ventricular end systolic volume. *P < 0.05 compared with postoperative 2nd week; ^#^ *P* < 0.05 compared with the control group.

### 3.6 Serum TNF-α, BNP, cTnT and LDH levels

**Figure 4:**
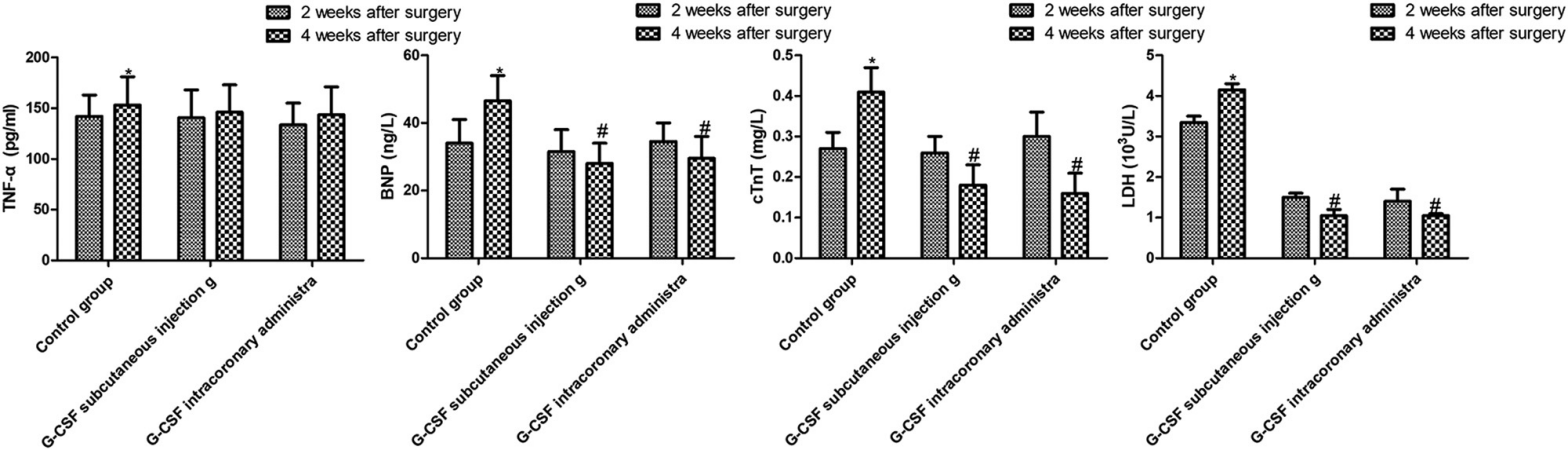
Serum TNF-α, BNP, cTnT and LDH levels. *P < 0.05 compared with postoperative 2nd week; ^#^ *P* < 0.05 compared with the control group.

Compared with 2 weeks after surgery, both G-CSF treatments significantly reduced the serum BNP level after 4 weeks of treatment (*P* < 0.05), but the level of the control group hardly changed. Compared with 2 weeks after surgery, the level of LDH in the control group increased significantly 4 weeks after surgery whereas decreased significantly after G-CSF treatment. The level of LDH in the G-CSF treatment group 4 weeks after surgery was significantly lower than that of the control group. After mobilization of G-CSF, the levels of TNF-α were significantly higher than those in the postoperative 2nd week, but the three groups had similar levels 4 weeks after surgery. In the postoperative 2nd week, the levels of cTnT in the peripheral blood of rats treated with G-CSF were lower than those before treatment (Figure 4).

### 3.7 Cardiac pathological examination

**Figure 5:**
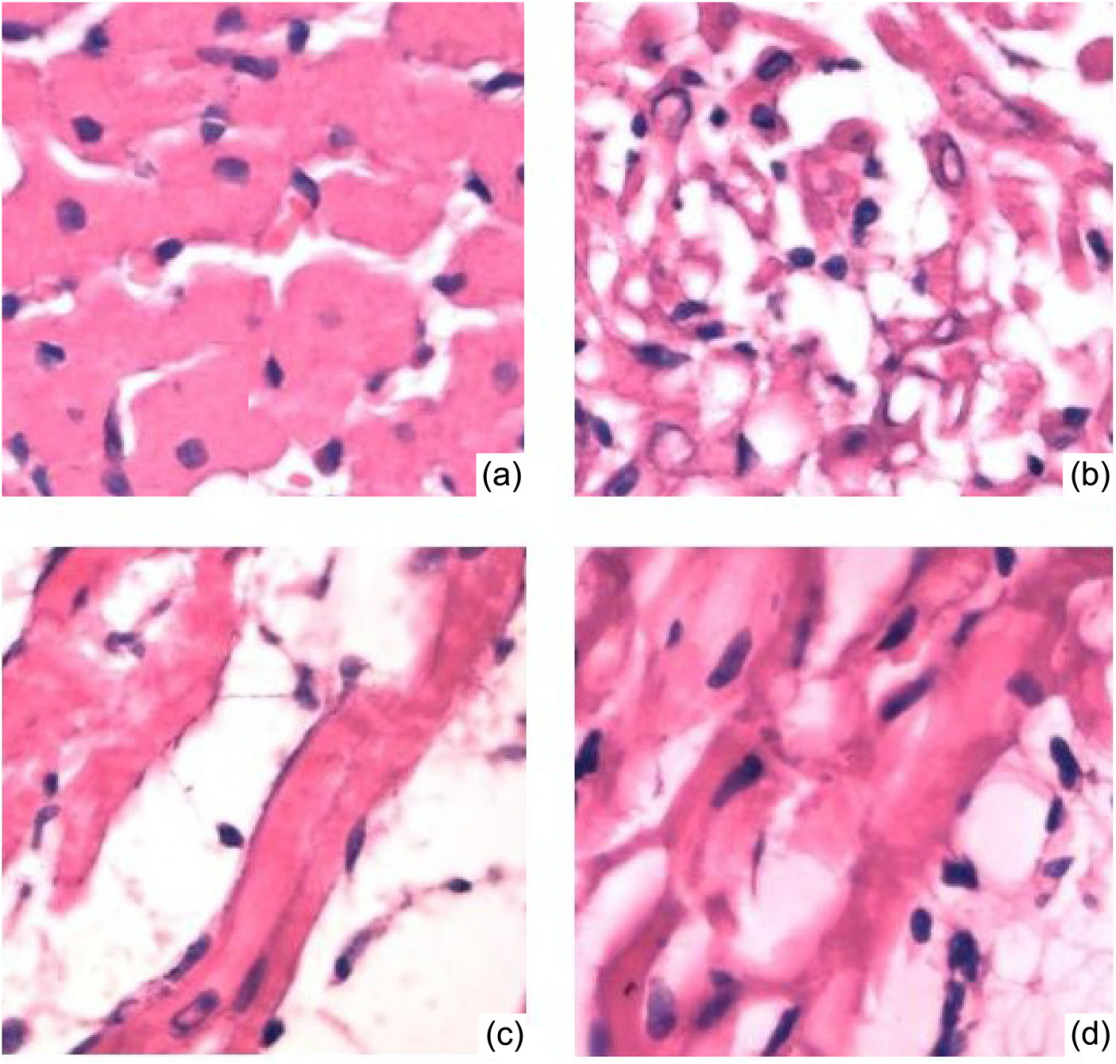
HE staining results of myocardial tissue 4 weeks after treatment. (a) Normal myocardial tissue. (b) Necrotic myocardium in the control group (sheet-like necrosis of myocardial tissue and local inflammatory cell infiltration). (c) Myocardial tissue of the subcutaneous G-CSF injection group (without sheet-like necrotic myocardium or interstitial fibrosis). (d) Myocardial tissue of the intracoronary G-CSF injection group (without sheet-like necrotic myocardium or interstitial fibrosis).

**Figure 6:**
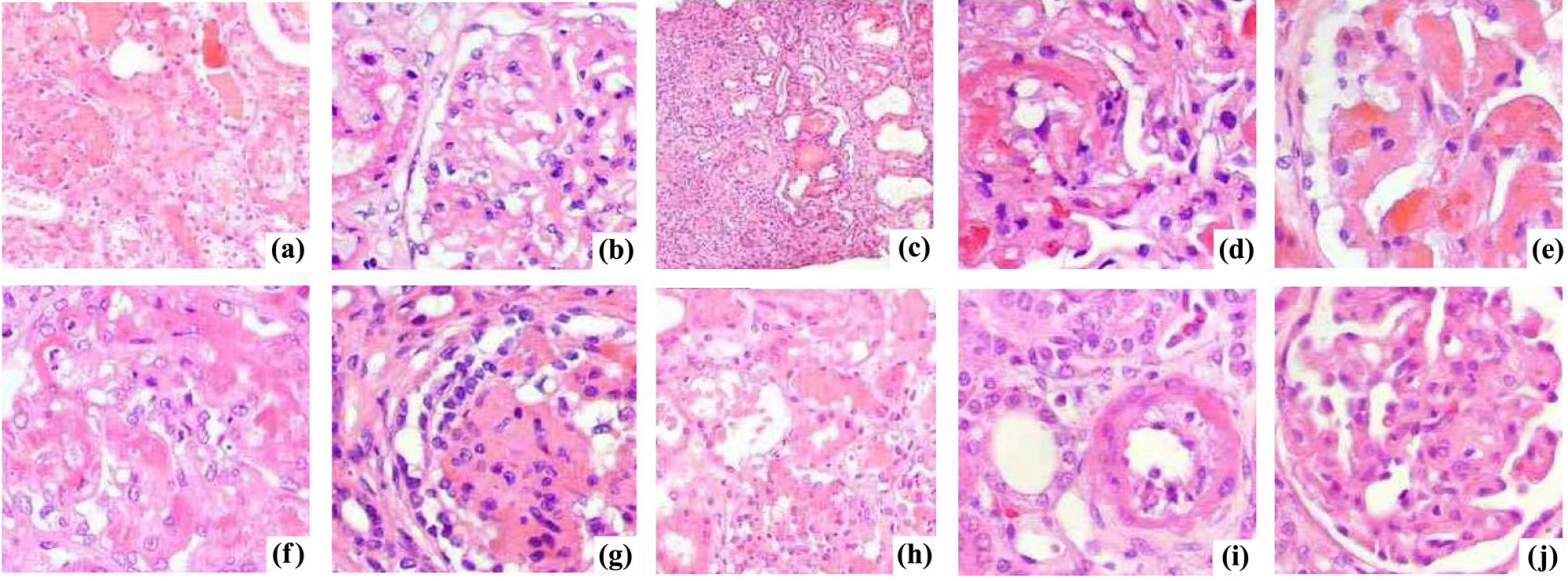
HE staining results of liver, kidney, lung, spleen and lymph node tissues. (a–e) Tissues of the control group. (f–j) Tissues of the intracoronary G-CSF injection group. Intracoronary injection of G-CSF did not cause the formation of new blood vessels in other major organs besides the heart, or new tumors.

Four weeks after surgery, the percentages of intimal hyperplasia in the intracoronary G-CSF injection group and subcutaneous G-CSF injection group were significantly lower than that of the control group (*P* < 0.001). The TNF-α levels in 30 rats were significantly positively correlated with the degree of myocardial damage (*r* = 0.437, *P* < 0.05). There was no significant difference between the former two groups. Myocardial HE staining showed that intracoronary injection of G-CSF also relieved myocardial necrosis (Figure 5). HE staining of tissues of the liver, kidney, lung, spleen and lymph nodes proved that G-CSF mobilization did not cause adverse reactions to other tissues outside the myocardium (Figure 6).

### 3.8 G-CSF mobilization and angiogenesis

**Figure 7:**
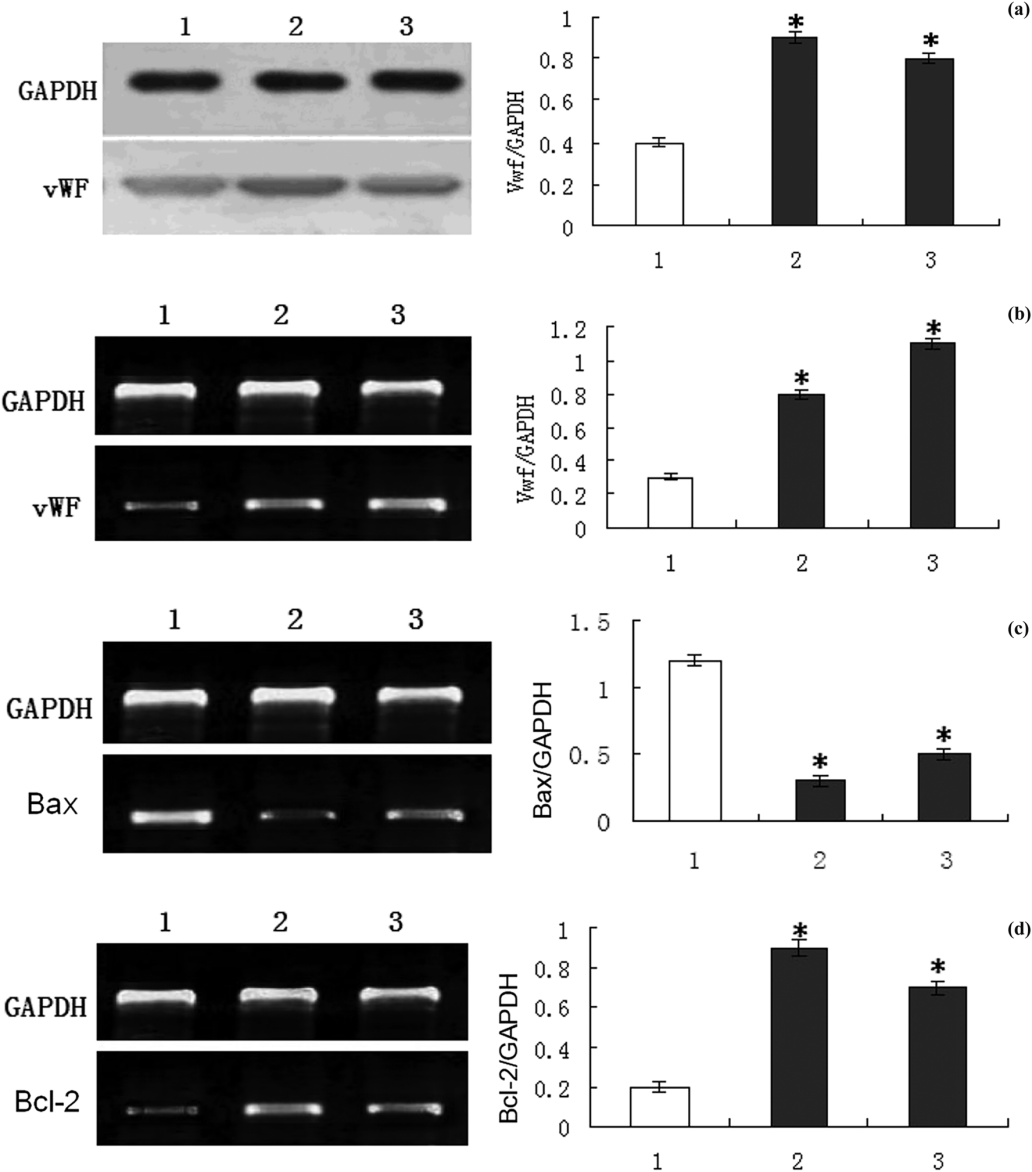
Effects of G-CSF mobilization on vWF, Bax and Bcl-2 protein and/or mRNA expression levels in infarct border zone. (a) Western blotting results of myocardial vWF protein expression levels. (b) RT-PCR results of myocardial vWF mRNA expression levels. (c) RT-PCR results of myocardial Bax mRNA expression levels. (d) RT-PCR results of myocardial Bcl-2 mRNA expression levels. (1) Control group, (2) subcutaneous G-CSF injection group and (3) intracoronary G-CSF injection group. *P < 0.05 compared with the control group.

The expression levels of vWF protein and the corresponding mRNA in the infarcted area were determined by Western blotting and RT-PCR, respectively. The levels of vWF protein and the corresponding mRNA in the control group were very low 4 weeks after surgery, whereas those of the subcutaneous G-CSF injection group and the intracoronary G-CSF injection group significantly increased (*P* < 0.05), suggesting that the number of new vessels in the infarct border area increased after G-CSF mobilization, but the two treatment groups had similar vWF expression levels (Figure 7a and b). The vWF mRNA levels in 30 rats were significantly positively correlated with CD34+ cell count and CD34+/CD133+ cell ratio (*r* = 0.657, 0.709, *P* < 0.05).

### 3.9 G-CSF mobilization and myocardial cell apoptosis

The TUNEL assay showed that the numbers of apoptotic cardiomyocytes in the infarct border zone of subcutaneous G-CSF injection group and intracoronary G-CSF injection group were lower than that of the control group 4 weeks after surgery (*P* < 0.05), but the numbers of the former two groups were similar. DNA ladder marker was used to identify the amplified bands at 223, 327 and 597 bp, respectively. The band positions were consistent with the theoretical ones of Bax, Bcl-2 and GAPDH. In the postoperative 8th week, the expression levels of Bcl-2 mRNA in the subcutaneous G-CSF injection group and intracoronary G-CSF injection group were higher than that of the control group, whereas the Bax mRNA expression levels of the former two groups were lower (*P* < 0.05). The two G-CSF treatment groups had similar Bax and Bcl-2 mRNA expression levels (Figure 7c and d).

## 4 Discussion

Death from ischemic heart disease accounts for 50% of all deaths from heart disease and 40% of all deaths from heart failure [10]. At present, an effective treatment for ischemic heart disease is still lacking. Heart transplantation may be helpful for patients with end-stage heart disease, but its clinical application is limited due to high medical cost, lack of donors and complications of the immune system. Recently, it has been demonstrated that stem cell transplantation can significantly improve cardiac function, reduce infarct size, increase local myocardial perfusion [11] and improve clinical symptoms, which has become a new approach for the treatment of heart failure [12]. This study confirmed that in addition to the conventional subcutaneous injection route, intracoronary injection of G-CSF was also safe and effective for treating CIHD. G-CSF can improve the left ventricular systolic function, reduce the area of myocardial injury and increase myocardial perfusion by reducing myocardial cell apoptosis in ischemic area and promoting local angiogenesis, thereby improving the cardiac function upon CIHD [13]. LDH is the most important marker for cell necrosis [14,15,16]. In this study, the level of LDH in the control group increased significantly 4 weeks after surgery in comparison with 2 weeks after surgery, whereas decreased significantly after G-CSF treatment. The level of LDH in the G-CSF treatment group 4 weeks after surgery was significantly lower than that of the control group.

G-CSF is a powerful mobilizing agent for bone marrow HSCs, which can simultaneously mobilize bone marrow HSCs, mesenchymal stem cells and endothelial stem cells to release them into the peripheral blood [17]. G-CSF can prevent ventricular remodeling after acute myocardial infarction and improve myocardial ischemia, which may be ascribed to direct activation of the Jak2/STAT3 pathway and promotion of local revascularization [18]. However, there are few studies on the treatment of CIHD by using G-CSF up to now [19]. Achilli et al. found significant differences in patients’ baseline conditions of inclusion, and the results were not the same [20]. Lipinski et al. performed a meta-analysis for the effects of intracoronary cell therapy on the left ventricular function in the setting of acute myocardial infarction and found that the subjects receiving this therapy had significantly improved LVEF, as well as reduced infarct size and end-systolic volume, accompanied by decreased end-diastolic volume [21]. This therapy was also related to significantly reduced recurrent acute myocardial infarction and trends toward decreased death, rehospitalization due to heart failure and repeated revascularization. This meta-analysis confirms the beneficial effects of this novel therapy on event-free and overall long-term survival. Additionally, Gupta et al. reviewed data accumulated through 10 years of clinical trials and proved that gene therapy had an acceptable safety profile [22]. Nevertheless, more rigorous phase II and III clinical trials are still in need to verify that angiogenic agents are beneficial to patients with ischemic disease. Whether G-CSF, which contributes to the restoration of cardiac function after acute myocardial infarction, has the same effect on CIHD has not been confirmed [23]. Therefore, before the large-scale use of G-CSF in clinical practice, it is necessary to evaluate the safety, efficacy and possible mechanism of G-CSF treatment at the animal level. In this study, we used the ameroid constrictor ring to insert the proximal segment of LCX branch. After the animal model of CIHD was successfully established, G-CSF was injected subcutaneously or directly into the coronary artery for 3 consecutive days. Two weeks later, neovascularization in the infarcted marginal area increased significantly, suggesting that G-CSF may improve the ischemic heart function by facilitating neovascularization. Meanwhile, there was no significant difference in the vWF expression between subcutaneous injection and coronary injection groups.

G-CSF can cause the mobilization of bone marrow stem cells including EPC to the peripheral blood [24]. This study also confirmed that the CD34+ cell count and CD34+/CD133+ cell ratio in the peripheral blood increased after G-CSF mobilization, being closely related to the enhancement of neovascularization. Moreover, the number of apoptotic cardiomyocytes decreased significantly after G-CSF treatment, and the increase in the number of new blood vessels may be related to the decrease in the number of apoptotic cardiomyocytes, which ultimately inhibited myocardial fibrosis and prevented the decline of cardiac function. Although we did not find herein that G-CSF treatment significantly reduced the number of apoptotic cardiomyocytes, the number of new blood vessels still increased, so the facilitation of neovascularization induced directly or indirectly by G-CSF attenuated myocardial cell death in animals with CIHD. Considering that G-CSF mobilization may lead to a hypercoagulable state, high-dose G-CSF mobilization may aggravate atherosclerosis.

## 5 Conclusions

Coronary G-CSF injection did not promote the proliferation of distant organ cells or the increase of neovascularization, and no adverse reactions such as death, myocardial infarction and heart failure caused by coronary injection were found, which preliminarily proved that the method was safe and feasible. Meanwhile, MRI and SPECT confirmed that the effect of G-CSF injection via the coronary artery was similar to that of subcutaneous injection in improving the cardiac function. In addition, the dose of G-CSF was reduced, which suggests that, it is possible to mitigate the adverse reactions caused by the considerable use of G-CSF. Regardless, the number of animals in this study is not large, so the findings should be further confirmed by experiments using more animals and clinical studies.
